# Regional health workforce monitoring as governance innovation: a German model to coordinate sectoral demand, skill mix and mobility

**DOI:** 10.1186/s12960-016-0170-3

**Published:** 2016-11-28

**Authors:** E. Kuhlmann, O. Lauxen, C. Larsen

**Affiliations:** 1 Institute for Economics, Labour and Culture, Goethe University Frankfurt, Frankfurt, Germany; 2 Medical Management Centre, LIME, Karolinska Institutet, Stockholm, Sweden

**Keywords:** Regional health workforce monitoring, Health workforce governance, Skill mix, Cross-border mobility, Transsectoral governance, Policy implementation, Germany

## Abstract

**Background:**

As health workforce policy is gaining momentum, data sources and monitoring systems have significantly improved in the European Union and internationally. Yet data remain poorly connected to policy-making and implementation and often do not adequately support integrated approaches. This brings the importance of governance and the need for innovation into play.

**Case:**

The present case study introduces a regional health workforce monitor in the German Federal State of Rhineland-Palatinate and seeks to explore the capacity of monitoring to innovate health workforce governance. The monitor applies an approach from the European Network on Regional Labour Market Monitoring to the health workforce. The novel aspect of this model is an integrated, procedural approach that promotes a ‘learning system’ of governance based on three interconnected pillars: mixed methods and bottom-up data collection, strong stakeholder involvement with complex communication tools and shared decision- and policy-making. Selected empirical examples illustrate the approach and the tools focusing on two aspects: the connection between sectoral, occupational and mobility data to analyse skill/qualification mixes and the supply–demand matches and the connection between monitoring and stakeholder-driven policy.

**Conclusion:**

Regional health workforce monitoring can promote effective governance in high-income countries like Germany with overall high density of health workers but maldistribution of staff and skills. The regional stakeholder networks are cost-effective and easily accessible and might therefore be appealing also to low- and middle-income countries.

## Background

It is increasingly becoming clear that better health workforce governance can make a difference to health system performance and policy implementation [1–5] and this brings monitoring into sight as a backbone of informed policy-making. Over recent years, data on health human resources have significantly improved in the European Union (EU) member states and internationally [6–11]. Yet data do not bring about change in the health workforce if not connected systematically to policy-making and implementation. A review of global developments found that ‘countries tend to generate basic [human resources for health] HRH supply and deployment data’, while ‘few seem to be explicitly using this information for making workforce decisions’ [12]. A recent policy evaluation of human resources for health recommendations in resource-poor settings revealed that ‘action is more likely to take place when … stakeholders are involved in the formulation and the implementation of policy changes’ [13]. Available research highlights the importance of governance for successful health workforce policy and the need for innovation [14, 15].

The present case study contributes a practice example and in-depth knowledge on *how* to make policy action through stakeholder involvement happen in a resource-rich setting. We apply an approach developed in EU regional labour market monitoring [16, 17] to the health workforce and explore the innovative governance capacity. The case study introduces a regional monitor developed in the Federal State of Rhineland-Palatinate (RLP) in Germany. More specifically, we seek to illustrate how a stakeholder-driven procedural approach promotes comprehensive data collection and how this feeds in a ‘learning system’ of governance to better match demand- and supply-side developments. The capacity to ‘learn’ is promoted through interactive discussions and evaluations. The model connects bottom-up and top-down governance based on mutual learning and shared decision-making of plural stakeholders. Our analysis is informed by multi-level governance approaches, including the substance and the hierarchal levels of decision-making [14, 18–21]. The focus is on nurses as the largest group, which is a major source of innovation and much in need of sustainable governance [22–24].

The literature identifies a number of problems and gaps in health workforce monitoring, which include an overall lack of needs-based approaches [25, 26] and persisting ‘silo’ approaches based on occupational categories and professional interests [20]. As Dussault and Dubois have highlighted some time ago, ‘the integrated, interdependent and systemic nature of the different components’ of the health workforce is still poorly reflected in planning and management [27]. Only little progress can be observed. Despite growing demand for improved skill mix, task-shifting and coordination [24, 28–32], many monitoring systems furthermore focus on planning and on doctors [23–37]. A further challenge is geographical imbalances between and within countries and growing mobility, including qualitatively new challenges of temporary and circular mobility, cross-border commuters and the internationalization of education and training [38–41]. Here, the EU single labour market and recognition of qualifications is a particular case in point [6, 9, 11, 42–49].

The major problem of the health workforce is poor governance, which makes it a health system problem [5]. Buchan has argued some time ago for health system-based solutions in relation to the shortage of nurses: ‘Until this is understood, and we make better use of the available evidence, we are doomed to endlessly repeat a cycle of inadequate, uncoordinated, obsolete and often inappropriate policy responses’ ([50], p. 458; see also [27, 51]). A recent policy analysis recalls that ‘the need to pay attention to components of systemic and intersectoral governance is becoming increasingly clear’ ([19], p. 130). So there is no shortage of problem diagnoses, and ‘therapeutic advice’ and measurements have also significantly improved [1, 26, 52–56]. Yet little is known on how to make better health workforce governance happen.

The aim of this case study is to explore how monitoring may serve as a governance tool to improve policy-making and implementation. We begin with introducing the German context and then provide information on the RLP regional health workforce monitor focusing on three dimensions: the conceptual model, the empirical data and the governance dimensions to make implementation happen. Major results are discussed, and the capacity of regional health workforce monitoring as a governance tool is explored using a multi-dimensional governance framework introduced by Greer et al., WHO European Observatory for Health Systems and Policy [3].

## Case presentation

### Regional health workforce monitoring in Rhineland-Palatinate, Germany

The regional health workforce monitoring was introduced by the Federal Government in 2002 as a ‘Branch Monitor’, including 36 municipalities with 3.99 million (in 2011) inhabitants in the Federal State of Rhineland-Palatinate. The monitor uses methods developed by the European Network on Regional Labour Market Monitoring (http://www.regionallabourmarketmonitoring.net/), which combines administrative data and ‘local’ bottom-up collected stakeholder knowledge to reduce skills mismatches [16, 57]. The labour market monitoring approach was initially transferred to the health sector to support evidence-based health workforce forecasting and planning; it is based on a demand–supply model connected to population development and needs. Hence, the monitor has continuously expanded and is now emerging as a complex governance tool.

#### The regulatory and occupational contexts

The German healthcare system is decentralized and federalist, based on a Bismarckian-type corporatist governance model of partnership between the statutory health insurance (SHI) and the physicians’ associations. Medical power is strongly integrated in the institutions of the healthcare system [58, 59]. In the main regulatory body, doctors represent the provider side and sickness funds the user side. Together, they form the joint self-administration, which counts as the ‘Achilles heel’ of the SHI system and cornerstone of partnership governance. Yet this model of conservative corporatist partnership governance constrains participation of nurses and other health professions, who do not have full self-governing rights, although some change is underway. In this situation, health workforce planning is fragmented and lacks coordination. Capacity planning for doctors is centralized with physicians’ associations as data providers; a major tool is a numerus clausus model, which is complemented by a decentralized mix of legal restrictions and incentive-based governance to reduce geographical imbalances and boost primary care [48, 49]. Healthcare assistants (primarily employed in ambulatory care by office-based physicians) are connected to physicians’ associations and supervised by doctors. For nurses, elder care nurses and other health professions/occupations, responsibility for planning and education is under the authority of the Federal States (http://www.kmk.org/fileadmin/Dateien/veroeffentlichungen_beschluesse/2015/2015_06_12-Fachkraeftesicherung-im-Gesundheitswesen.pdf). As a consequence, data sources are fragmented, workforce governance models for nurses less well developed than for doctors and comprehensive integrated models of monitoring lacking [60–62].

Germany falls in the group of OECD countries with high staffing levels—4.1 practising doctors and 13.0 practising nurses per 1000 population ([26], Figures 2.4 and 2.5]). However, individual hospital health workers perceive staff shortage as a serious problem [63] and geographic imbalances and maldistribution of skills may cause shortage especially in nurses and in remote and rural areas [23, 40]. Traditionally, Germany aimed for a self-sufficient health workforce, but demographic trends create a rapidly widening gap between demand and supply, affecting nurses and elder/long-term care (LTC) the most. In this situation, the percentage of foreign-trained professionals is increasing, especially in medicine (8.8% doctors) and to a lesser degree in nurses (5.1%) ([49], Tables 4.1 and 4.2]).

Labour market trends show contradictory developments. Cost-containment policies caused significant cuts in the number of hospital nurses, while staff levels of nurses expanded in LTC, and doctors faced an overall increase in numbers [64]. Contrary to most other OECD countries, Germany does not promote task-shifting policies from doctors to nurses [24, 30]. Also, academic training was introduced much later than in most other EU countries, and new roles, like nurse practitioner, are not established. It should be noted that the RLP Federal Government has long since supported health workforce policy and RLP is a forerunner in establishing a nursing chamber (*Pflegekammer*) (http://www.100prozent-pflegekammer.de). Within this context, a regional monitor project may bypass the health system blockades to integrated workforce governance, to some degree, through local stakeholder support.

#### The conceptual dimension: methods, tools and actors

The RLP monitor was established in 2002 as a labour market-based nursing monitor and has been continuously expanded since 2008, now including three occupational clusters, namely nurses/carers, therapists and assistant/allied health professions. The monitor comprises 18 health professions working in the hospital and elderly care/LTC sectors (in-patient and out-patient care providers) [60, 65] (for an example, see http://www.branchenmonitoring-rlp.de/gesundheitsfachberufe/download/Fragebogen_Altenhilfe.pdf). The focus is on middle-level to basic qualifications. Other health professions/occupations are considered, but currently not monitored, and doctors do not participate in the monitor.

Stakeholder involvement is a guiding principle of regional labour market monitoring [57] and has been embedded in the RLP monitor since its early days. It received a boost in 2012 through the development of a formalized governance structure that connects top-down (represented by the Federal Government) hierarchical governance and bottom-up network governance through a board of plural stakeholders and subordinated working groups (for more information, see Table 3). The emergent participatory governance model systematically connects monitoring and policy-making. The next transformation of monitoring into a governance tool followed in 2013 with the introduction of a module for the so-called Greater Region, comprising Luxembourg, France and Belgium and the two German Federal States RPL and Saarland [39, 66]. Here, the aim was to establish cross-border collaboration and improve information through local knowledge, especially on ‘atypical’ mobility and commuters. For instance, German citizens may register in a neighbour country and commute to their work place in Germany or vice versa.

The monitor is based on three pillars—data collection, communication and decision-making—which are systematically connected in two ways. Institutionally, a network-based board of stakeholders serves as an arm’s-length organization furnished with support from the Federal Government and the stakeholders. Conceptually, participatory governance and a process-based approach create a channel for information from monitoring into the policy process and back again, thus promoting accountability and transparency of decision-making. The monitor follows an integrated approach [20, 21] that connects health and education systems, hospital and LTC sectors, different organizations/providers and different health professions and qualifications. The operational governance tools are presented in Table 1.

**Table 1:** Conceptual dimensions and operational governance tools of regional labour market monitoring

Dimensions of monitoring	Governance tools
Information: data collection and quality improvement	• Efficient/‘intelligent’ data collection through the matching of primary data collection and secondary sources, representative statistics and qualitative explorative expert knowledge, and time series • Expert review and multiple stakeholder perspectives to improve quality of data • Expansion and adaption of data source and categories • Standardization of workforce categories
Communication: stakeholder involvement and networking	• Joint assessment of data and knowledge • Shared decision-making based on transparent criteria • Collaboration and coordination between stakeholders, sectors and professional groups • Pioneering cross-border collaboration and coordination • Developing governance structures bottom-up to reduce institutional gaps and transaction costs
Decision: policy-making and implementation	• Building structure and formalizing bottom-up decision-making and stakeholder involvement through establishment of a board of (a broad range of) stakeholders • Developing participatory governance • Establishing cross-border stakeholder groups and decision-making bodies • Connecting health and education systems to improve policy implementation • Developing thematic working groups to facilitate implementation • Defining success criteria and target setting • Evaluating policies to promote transparency

Source: own analysis based on published information, internal documents and personal communication from the RLP monitor

Characteristically, the three pillars of monitoring combine classic tools of planning (such as statistical data and analysis) and novel operational tools of actor-centred governance in order to promote policy, implementation and evaluation. Examples of this will be illustrated in more detail in the next sections.

#### The empirical contribution: selected illustrative results of an integrated monitoring

Regional monitoring data provide in-depth information on the (mis-)matches between supply and demand and the skill mix trends or, more precisely, the qualification mix. For our purpose, the two groups of nurses—registered nurses (RNs) and assistant nurses (ANs)—are selected from the sample of 18 professional groups for an empirical illustration. It should be noted that, in 2010, RLP had not introduced academic nursing education (mirroring the German situation). Since then, pilots have been established, but data are not yet available. Our analysis is based on administrative data gathered in the RLP health workforce monitor in 2011 and amended with most recent mobility and evaluation data. A second wave of data collection is currently in progress, but may not be available publicly within the next year. Table 2 below summarizes the workforce trends and composition of nurses in relation to sectoral composition, qualification mix and mobility.

**Table 2:** Workforce trends and qualification mix of nurses in different settings

Category	Registered nurses^a^	Assistant nurses^b^	Developments in relation to qualification mix
Workforce trends (1999–2011)	22.5% increase	7.5% increase	% increase in RNs is 3× higher than in ANs
Qualification mix trend 1999–2011	7.9 RNs:1 AN (in 1999)		Relevant increase in qualification overtime
	9.0. RNs:1 AN (in 2011)		
Qualification mix per sector, 2011			The share of RNs per AN is nearly five times higher in the hospital sector than in LTC
Hospital	22.4 RNs:1 AN		
LTC	4.9 RNs:1 AN		
Nursing staff per sector^c^			Hospital sector is better resourced in numbers and qualifications
Hospital (54%)	95.7%	4.3%	
LTC (46%)	83.2%	16.8%	
Mobility (% of total)			Mobility is overall low; outflow of RNs 6.5× higher than inflow, outflow–inflow of ANs is more balanced
Outflow^d^:Inflow^e^	1.3%:0.2%	0.7%:0.6%	

Sources: RLP monitoring data, head counts

^a^RNs include at least 3 years educated nurses, elder care nurses and paediatric nurses

^b^ANs include nurse assistants and elder care assistants, with 1-year education according to Federal State RLP regulation

^c^Hospital sectors include the following: hospitals, mental healthcare clinics and rehabilitation clinics; LTC sector includes the following: ambulatory care and in-patient care provider organizations

^d^Outflow (focused on Luxembourg, as mobility to the other border countries can be neglected): percentage of nurses with German citizenship working in hospitals in Luxembourg of all nurses in RLP

^e^Inflow from Belgium, France and Luxemburg, 2011

The analysis reveals a shift towards higher qualification of nurses over time; the percentage of RNs is growing three times faster compared to ANs. To some degree, this result seems to mirror EU comparative research, which found an overall increase in staffing levels but slower expansion in the occupational groups at the basis [64]. This trend is also relevant in RLP but must be seen in the context of an overall very high qualification level of nurses. ANs count for only 10% of the total nursing staff, and 77% are working in the LTC sector, yet even in LTC, the numbers of RNs remain nearly five times higher than those of ANs.

In relation to cross-border mobility, the outflows from RLP to Luxembourg are higher than the inflows and mobility is more relevant in the group of RNs than for ANs. In 2012, German citizens accounted for 12.9% of RNs and 4.7% of ANs of the nursing workforce in Luxembourg. One important reason is the higher salaries in Luxembourg. When looking at the inflows to RLP, numbers are overall low, and most importantly, the majority of these nurses are commuters—German citizens living outside the country and coming in for work.

Table 3 provides information on the staff levels (in absolute and relative numbers) and matches between demand and supply. Currently, 622 provider organizations are participating in the monitor, including 62.4% of hospitals and 59.9% of LTC providers in RLP. These organizations deliver data on job vacancies, measured as head counts, which are set in relation to the new annual supply, based on representative data of the RLP unemployment offices on unemployed nurses fit for practice and data on nursing graduates from the statistical office of RLP.

**Table 3:** Staff levels and supply–demand matches in Rhineland-Palatinate

Category	Total nurse workforce, head counts	RN workforce	AN workforce	Qualification mix
Practising nursing staff	39 390	35 453	3 937	9.0:1
Hospital (54%)	21 449	20 532	917	
LTC (46%)	17 941	14 921	3 020	
New supply^a^	3 278	2 659	619	4.3:1
New supply in % of practising nursing staff	8.3%	7.5%	15.7%	Relatively higher increase in ANs
Demand total and per sector^b^	6 182	4 925	1 257	3.9:1
Hospital	1 498	1 462	36	
LTC	4 684	3 463	1 221	
Demand in % of practising nursing staff, total and sector	15.7%	13.8%	31.9%	Relatively higher demand for ANs and LTC
Hospital	6.9%	7.1%	3.9%	
LTC	26.1%	23.2	40.4%	
Shortage in % of practising staff total and per qualification	−2 904	−2 266	−638	Relatively higher shortage of ANs
	7.4%	6.6%	16.2%	

Source: RLP Regional Health Workforce Monitoring, own calculations

^a^Head counts, based on education outflow + unemployment data, 2010

^b^Head counts, based on data collected from the provider institutions, 2010

The results reveal an overall strong mismatch between supply and demand, which is to a high degree an effect of strongly increasing demand for LTC and for ANs. This sector- and qualification-specific pattern of demand mirrors the changing population situation. Statistics show an overall decreasing number of inhabitants in RLP since 2004 and the typical German picture of a rapidly ‘ageing society’ (www.statistik.rlp.de). More specifically, we can observe an overall decrease in population, which is strongest in the labour market active group of 20- to 65-year-old citizens (a 17% decrease). An adverse trend occurs with a 43% increase in population in the age group 65+ and 56% in the group older than 80 years (www.statistik.rlp.de). This situation calls for an adequate expansion in LTC services and, accordingly, for more ANs to provide basic services to patients. Thus, different needs for care in LTC and hospitals may create changes in the skill mix to some degree, although a quota of fully qualified nurses is legally defined. The selected examples highlight that an initial demand–supply monitoring model based on strong stakeholder involvement may to some extent respond to changing needs of the population, yet further discussion is necessary to define ‘needs’ and the ‘right’ mix of skills. The next section explains how monitoring is connected to policy and implementation.

#### The governance dimension: facilitating policy and implementation

The major strength of the regional health workforce monitor is its capacity to provide reliable empirical data and improve coordination in fragmented institutional settings, like the German healthcare system. This makes monitoring an innovative governance tool to facilitate policy-making and implementation. One important issue is the connection of bottom-up and top-down policy-making that involves three major actors: the government, the board of stakeholders and an academic institution. Through this connection, different sources of power, knowledge and agency combine and maximize the opportunities of improving health workforce governance. Stakeholder involvement is a particular case in point: it strengthens accountability and collaboration with provider organizations. The more organizations are willing to feed data into the monitoring system and collaborate, the higher the reliability of planning and targets, and the effectiveness of policy implementation. Table 4 summarizes the major actors and processes, which create a ‘learning system’ of health workforce governance based on scientific evidence and expert knowledge of practice partners.

**Table 4:** Regional labour market monitoring as ‘learning system’ of policy actors and processes

Dimensions of monitoring	Actors and processes
Policy actors	• Representatives from educational institutions/schools • Professional associations • Unemployment insurance • Sickness funds • Old age care insurance • Federal Government/ministries • Researchers
Policy processes	• Building networks and channels to improve coordination • Connecting government, stakeholder board and research to enable shared decision-making • Reducing interest-driven strategies of professional groups through developing shared goals and identities to improve integrated policy-making • Providing research-based data to improve evidence-based decision-making
Policy implementation and evaluation	• Connecting top-town (government) regulatory power and bottom-up stakeholder agency • Using stakeholder participation to strengthen accountability and support of provider organizations and institutions • Establishing evaluation of targets and policies
Learning system/governance improvements	• Revising targets and policy according to evaluation data • Establishing transsectoral and trans-border governance • Including new stakeholders where relevant

Source: authors’ own table

The evaluation of the targets for RN education provides an empirical example of the ‘learning system’. In 2011, when the monitoring model was less mature, there were more than 1100 vacancies in nursing [65]. From 2012 to 2015/2016, the education of RNs increased continuously and the shortage was reduced to about 700 vacancies following the implementation of a more complex decision-making procedure. The next step will be to revise the targets and critically discuss the policy options. Due to the demographic developments in RLP (and others parts of Germany), it will increasingly become necessary to connect education-based capacity planning to a set of health workforce governance tools to close a widening gap between demand and supply. This includes improved recruitment and retention strategies, organizational restructuring and changes in the mix of skills and qualifications [67–69].

### Discussion

This case study shows how regional health workforce monitoring can add new knowledge on developments especially in areas not well covered by other systems, like skill/qualification mix, sectoral dynamics and mobile workers in border regions. It reveals opportunities for connecting demand–supply and needs-based monitoring approaches through strong stakeholder involvement and systematic fine-grained regional data collection. The most important contribution is the capacity to innovate health workforce governance by disrupting a cycle of ineffective health workforce policy [2, 50]. Following Greer et al., ‘[T]he way decisions are made and implemented is crucial to the success of policies for health and to achieving the desired health outcomes in any system. Policies with a strong evidence base and financial resources can fail in implementation because of governance, while governance can shape the likelihood that good policies are adopted’ ([19], p. 130).

A governance framework recently introduced by the WHO European Observatory for Health Systems and Policies [14, 15] may help to assess the strength (and weakness) of the regional health workforce monitor. This framework includes five components: transparency, accountability, participation, integrity and capacity (TAPIC) ([14], p. 3), which are defined as follows. ‘Transparency is how and how much decisions and their grounds are made known. Accountability is explanation and sanction – who can effectively demand an explanation and sanction an action? Participation is the participation of those affected by a decision in the decision-making. Integrity is the establishment of non-corrupt, institutionalized organization. Capacity is policy capacity – the existence of expertise on policy formulation, implementation and evaluation’ ([15], p. 106).

Against this backdrop, ‘participation’ provides the overarching conceptual and strategic guidance of the RLP monitor, which is operationalized as strong stakeholder involvement on all levels and areas of decision-making. Greer et al. illustrate the practical dimensions of governance, raising questions like ‘who is in the room when the decision is made, and will they support the policy in practice?’ ([15], p. 105) These are precisely the underlying questions of stakeholder involvement in the RLP monitoring project that can explain an increased benefit through an expansion of relevant players in the field. Another success factor is ‘policy capacity’ operationalized through formalized structures to connect top-down and bottom-up decision-making (such as boards), through indicators to measure policy implementation (job vacancies, educational intakes, etc.), procedural target setting (e.g. education and training capacities) and policy revision. ‘Transparency’ and ‘integrity’ are also important components of the RLP monitor as data are publically available and the stakeholder network reinforces the need to negotiate and justify political, professional and provider interests against the population needs. The commitment to shared decision-making may contribute to accountability, although the opportunities are not fully explored and go beyond regional capacity; here, health system-based changes in Germany are called for to strengthen needs-based approaches and patient involvement.

Our analysis has demonstrated *how* the components may be connected to improve governance. Stakeholder involvement and a formalized procedural approach make for the ‘grease’ in the monitor system to reduce transaction costs of policy implementation and enable innovative health workforce governance through a ‘learning system’ of policy-making. In this regard, the RLP monitor complements evaluations in resource-poor settings, suggesting that ‘participation’ may be an effective governance tool in different contexts [12, 13, 70]. Other advantages include in-depth knowledge available on the regional level, promoting ‘intelligent’ data use. Especially useful is a systematic connection between quantitative statistical data, focused on the occupational structure and labour market development, and qualitative expert information of the various stakeholders, which add valuable knowledge on the substance of health workforce development. There is also a ‘geographical bonus’ due to shorter distances and regionally established communication channels, which may to some extent bypass fragmented and interest-driven governance structures. These conditions provide cost-efficient solutions for complex integrated monitoring systems to inform health workforce policy and evaluate the effects.

### Limitations

The network-based character of regional health workforce monitoring is both a strength and a weakness. While a weak institutionalization provides greater flexibility and facilitates innovation, the outcomes may be less sustainable and highly context dependent. Most importantly, in order to achieve sustainability and strengthen the transformative potential of a regional approach, the monitoring system must be flanked by, and connected to, change in the legal frameworks and other governance reform on the national level. Furthermore, the model cannot reduce the gaps between more centralized capacity planning for doctors and the decentralized, federalist models for other health workers, as doctors do not participate; inclusion of healthcare assistants would also be helpful especially for primary healthcare planning. Another important limitation is the inter-linkage of the model with labour market approaches, which do not adequately reflect the role of organizations and health workforce management. This is especially relevant in relation to improvement of recruitment and retention strategies and a more effective use of staff, skills and qualification mixes [67, 68]. Also, the demand–supply-based monitoring model would benefit from further systematic connection to population needs, including discussing ways of defining needs, staffing levels and the composition of skills.

## Conclusions

The case study has set out to introduce regional health workforce monitoring as a tool for governance innovation. The illustrative examples reveal how monitoring can make a difference to health workforce governance. The core elements of this model comprise ‘intelligent’ data collection through connecting different sources, strong stakeholder involvement and a procedural approach to policy as a ‘learning system’ of decision-making. This monitoring model promotes capacity building for integrated health workforce monitoring and governance in an otherwise fragmented planning system, which is biased towards medical provider groups, while integration of nurses and other groups are constrained. The case study illustrates how health system deficiencies and governance gaps may be reduced to some degree through bottom-up driven innovation. Next to other German federal states and resource-rich countries, especially with federalist governance, the monitoring model may also be appealing to low- and middle-come countries, because of its cost-effective nature and easily accessible regional data and governance networks. Systematic transformation of the tools to the contexts of resource-poorer health systems may help to establish sustainable health workforce governance and universal healthcare coverage.
